# Artificial intelligence–assisted rectus femoris ultrasound vs. L3 computed tomography for sarcopenia assessment in oncology patients: establishing diagnostic cut-offs for muscle mass and quality

**DOI:** 10.3389/fnut.2025.1678989

**Published:** 2025-09-25

**Authors:** Juan José López-Gómez, Israel Sánchez-Lite, Pablo Fernández-Velasco, Olatz Izaola-Jauregui, Ángela Cebriá, Paloma Pérez-López, Jaime González-Gutiérrez, Lucía Estévez-Asensio, David Primo-Martín, Emilia Gómez-Hoyos, Eduardo Jorge-Godoy, Daniel A De Luis-Román

**Affiliations:** ^1^Servicio de Endocrinología y Nutrición. Hospital Clínico Universitario de Valladolid, Valladolid, Spain; ^2^Centro de Investigación en Endocrinología y Nutrición, Universidad de Valladolid, Valladolid, Spain; ^3^Servicio de Radiología, Hospital Clínico Universitario de Valladolid, Valladolid, Spain; ^4^DAWAKO Medtech SL, Parc Cientific de la Universitat de Valencia, Paterna, Valencia, Spain; ^5^Técnicas Avanzadas de Desarrollo de Software Centrado en la Persona, Departamento de Informática, Universitat de Valencia, Burjassot, Valencia, Spain

**Keywords:** muscle ultrasound, sarcopenia, disease-related malnutrition, computed tomography, artificial intelligence

## Abstract

**Introduction:**

Sarcopenia is prevalent among oncological patients; its diagnosis remains challenging due to technical limitations. This study aims to compare an AI-assisted ultrasound (US) evaluation of the rectus femoris with the gold-standard L3 computed tomography (CT) scan and to establish diagnostic cut-off values for sarcopenia in oncologic patients.

**Methods:**

This cross-sectional observational study assessed body composition in oncology patients undergoing treatment, comparing two AI-assisted imaging modalities: L3 vertebral level CT and US of the rectus femoris. Muscle mass was quantified using Skeletal Muscle Area (SMA) and Lean Muscle Area (LMA) from CT, and Rectus Femoris Area (RFA) and Thickness (RFT) from US. Muscle quality was evaluated via skeletal muscle Hounsfield units (SM-HU) on CT, and muscle (Mi) and fat (FATi) percentages within the region of interest on US. CT served as the reference standard for diagnosing malnutrition, sarcopenia, and myosteatosis.

**Results:**

A total of 337 patients (58.8% male; mean age 69.7 (10.9) years) were analyzed, with malnutrition identified in 78.3%, including 8% with sarcopenia. Ultrasound measurements correlated with CT-derived muscle metrics, including RFA with SMA (*r* = 0.44; *p* < 0.01) and LMA (*r* = 0.47; *p* < 0.01). Muscle quality parameters from US showed a positive correlation between Mi and SM-HU (*r* = 0.27; *p* < 0.01), while FATi negatively correlated with SM-HU (*r* = −0.19; *p* < 0.01). Ultrasound-based cut-off points for diagnosing sarcopenia were established at 3.425 cm^2^ RFA (AUC: 0.628) and 1.180 cm RFT (AUC: 0.636) for men, and 2.845 cm^2^ RFA (AUC: 0.628) and 0.868 cm RFT (AUC: 0.636) for women; and ultrasound-based cut-off points for diagnosing myosteatosis were 46.77% for MiT (AUC: 0.640) and 41.20% for FATi (AUC: 0.623). Derived cut-off values demonstrated high negative predictive value for low muscle mass and high positive predictive value for myosteatosis.

**Conclusion:**

AI-enhanced rectus femoris ultrasonography is a feasible, non-invasive, and clinically relevant approach for sarcopenia assessment in oncology. AI-assisted ultrasonography showed moderate correlations with CT-derived muscle mass indices and reliably capture qualitative changes in muscle quality and fat infiltration. The AI heralds a new era of rapid, radiation-free body composition assessment.

## 1 Introduction

Disease related malnutrition (DRM) is a condition with a high prevalence of 60% among hospitalized patients with chronic diseases, with up to 10% of them becoming malnourished during their hospital stay (1). This situation worsens with disease progression and varies according to the treatment administered (surgery, chemotherapy, or radiotherapy) (2). It is associated with numerous adverse outcomes: a higher number of complications, more frequent and prolonged stays, poor tolerance for aggressive treatments, and lower survival rates (2). Early detection of malnutrition risk, along with the early initiation of medical nutritional treatment, may be linked to a reduction in the rate of complications and a decrease in the average length of stay (3). Furthermore, sarcopenia in oncologic patients who are about to undergo surgery is associated with poorer postoperative outcomes in conditions as closely linked to malnutrition as pancreatic or gastric cancer (4, 5).

A proper diagnosis of DRM and sarcopenia demands an approach that goes beyond simply measuring body weight or using related estimates like the Body Mass Index (BMI) (6). Specific imaging methods like magnetic resonance imaging (MRI), computed tomography (CT), and dual-energy X-ray absorptiometry (DEXA) are considered the “gold standard” for evaluating body composition, particularly for the measurement of muscle mass. In fact, these imaging techniques are most commonly used as diagnostic criteria for both Global Leadership Initiative on Malnutrition (GLIM) (6, 7) and European Work Group on Sarcopenia in Older People (EWGSOP2) (8). CT scans can assess skeletal appendicular muscle mass by analyzing a region of interest at the L3 vertebral level (9). Nevertheless, these imaging modalities are not typically used in routine clinical practice due to logistical challenges, as they often require extra imaging that exposes patients to some level of ionizing radiation.

In everyday clinical practice, clinicians often rely on more accessible and easily reproducible methods due to their simplicity and availability. Traditional anthropometry and bioelectrical impedance analysis (BIA) are examples of such methods (10). Muscle ultrasound has recently gained attention as a dynamic technique for evaluating both the quantity and quality of muscle tissue in targeted areas of the body (11, 12). Typically, the procedure involves taking a transverse image of the rectus femoris in the quadriceps, from which practitioners measure the muscle mass (area, muscle thickness…) and assess the muscle’s echogenicity as an indicator of its quality (11). The main challenge with this method is that scientific evidence supporting its use is still limited. Moreover, since most ultrasound studies have been conducted on healthy elderly populations, its role in assessing DRM remains to be fully validated (12). Disease-Related caloric-protein malnutrition EChOgraphy (DRECO) study showed cut-off points for sarcopenia with a diagnosis of low muscle mass established by an estimation through BIA in patients at risk of malnutrition (13).

Artificial Intelligence (AI) in radiological body composition assessment lies in accurately identifying the relevant anatomical regions and interpreting the imaging data (14). AI significantly enhances the evaluation of body composition on CT scans through automatic image segmentation. It distinguishes between different components-such as fat, muscle, and other tissues-providing both quantitative and qualitative metrics. This capability has paved the way for processes that not only extract quantitative features quickly but also assess qualitative characteristics of the images, transforming them into actionable data. This approach is a core element of the emerging field of radiomics (15). Muscle ultrasound segmentation and standardization can be challenging due to the observer’s influence when performing the segmentation. Recent studies have demonstrated that using artificial intelligence for image segmentation in patients with DRM can be at least as effective as human observers (16). Moreover, AI-based tools have enabled the standardization of muscle quality evaluations. These technologies facilitate the extraction of features from conventional ultrasound images, providing concrete measurements within a defined region of interest (ROI). They allow for a detailed analysis of muscle architecture and quality by assessing parameters such as echogenicity and image texture biomarkers.

The objective of this study is to compare the evaluation of the quantity and quality of rectus femoris muscle-as assessed by muscle ultrasound assisted by artificial intelligence (17), with the analysis of muscle mass and quality determined by the gold standard CT scan of L3. Furthermore, the study aims to establish cut-off points of muscle mass and quality by ultrasonography in sarcopenia in a sample of oncology patients at risk of malnutrition. To achieve the objective of this study, patients with oncological pathology undergoing active treatment (surgery, chemotherapy, or radiotherapy) were evaluated through a comprehensive nutritional assessment, including muscle ultrasound of the rectus femoris. Additionally, abdominal CT scans at the L3 level, performed for other clinical reasons, were selected. Both ultrasound and CT images were analyzed to assess their correlation and to explore the possibility of establishing ultrasound-based cut-off points for muscle quantity and quality parameters.

## 2 Methods

### 2.1 Study design

This is a cross-sectional observational study designed to compare body composition parameters assessed using two different techniques, with a maximum time interval of 2 weeks between assessments: CT imaging at the L3 vertebral level (CT-L3) and muscle ultrasound of the Quadriceps Rectus Femoris (QRF) in patients with oncological conditions. The study examines both muscle quantity and quality using two different AI tools based on U-Net system to analyze the imaging results.

Additionally, the research seeks to diagnose malnutrition and sarcopenia by evaluating commonly used muscle composition and function parameters. It aims to establish cut-off values for sarcopenia, referencing the gold standard: computed tomography (CT scans).

Upon obtaining informed consent and enrolling the patients in the study, a comprehensive medical history assessment was conducted, including personal background, disease progression, and nutritional history. Anthropometric measurements and muscle ultrasound evaluations were performed. Electronic medical records were reviewed to determine whether control CT scans for the oncological condition had been conducted, and muscle mass and quality at the L3 vertebral level was analyzed with a maximum time interval of 2 weeks between consultation assessment and CT image obtention.

Using the collected data, an initial descriptive statistical analysis was performed to assess the prevalence and nutritional status of the patients, comparing different body composition evaluation techniques. A further statistical analysis will be carried out to examine the diagnostic accuracy of muscle ultrasound compared to CT parameters, as well as to define cut-off points for sarcopenia components, such as low muscle mass and low muscle strength, and to compare quality parameters determined via CT attenuation.

### 2.2 Patients selection

Patients were recruited between January 2021 and March 2025. The patients met the following criteria: inclusion criteria: outpatients with oncological conditions undergoing treatment or pending curative treatment (surgery, chemotherapy, and/or radiotherapy) who attended the nutrition consultation due to high nutritional risk, and individuals over 18 years old; exclusion criteria: patients with oncological conditions managed in the Palliative Care Unit without surgical, radiotherapeutic, or oncological treatment, patients who did not undergo an extended CT scan, those with decompensated liver disease, patients with Chronic Kidney Disease stage IV or higher, and patients who did not sign the informed consent.

The study complies with all ethical considerations outlined in the Declaration of Helsinki, and informed consent will be obtained from all patients. This study was reviewed by the Ethics Committee for Research with Medicines (CEIm) of the Valladolid Areas and was approved under code PI-GR-24307-C on May 8, 2024. All patients included signed the informed consent.

### 2.3 Variables

#### 2.3.1 Anthropometric measures

The measured anthropometric variables included current body weight (kg), usual body weight (kg), height (m), body mass index (BMI), arm circumference (cm), calf circumference (cm), and the percentage of body weight loss. Height was measured with a calibrated height scale (SECA, Germany), while body weight was determined using digital scales (SECA, Germany) with subjects minimally clothed and barefoot. BMI was calculated as weight in kilograms divided by height in meters squared (kg/m^2^). The percentage of body weight loss was computed using the formula: [(usual weight-current weight)/usual weight] × 100.

#### 2.3.2 AI-based body composition computed tomography at L3 level (CT-L3)

Abdominal CT scans (General Electric Revolution, Cincinnati, OH, USA) was analyzed by AI-tool (ARTIS Development, Las Palmas Gran Canaria, Spain). This software features an intuitive interface and a semi-automatic labelling tool, allowing user adjustments for body mass segmentation.

**Figure 1:**
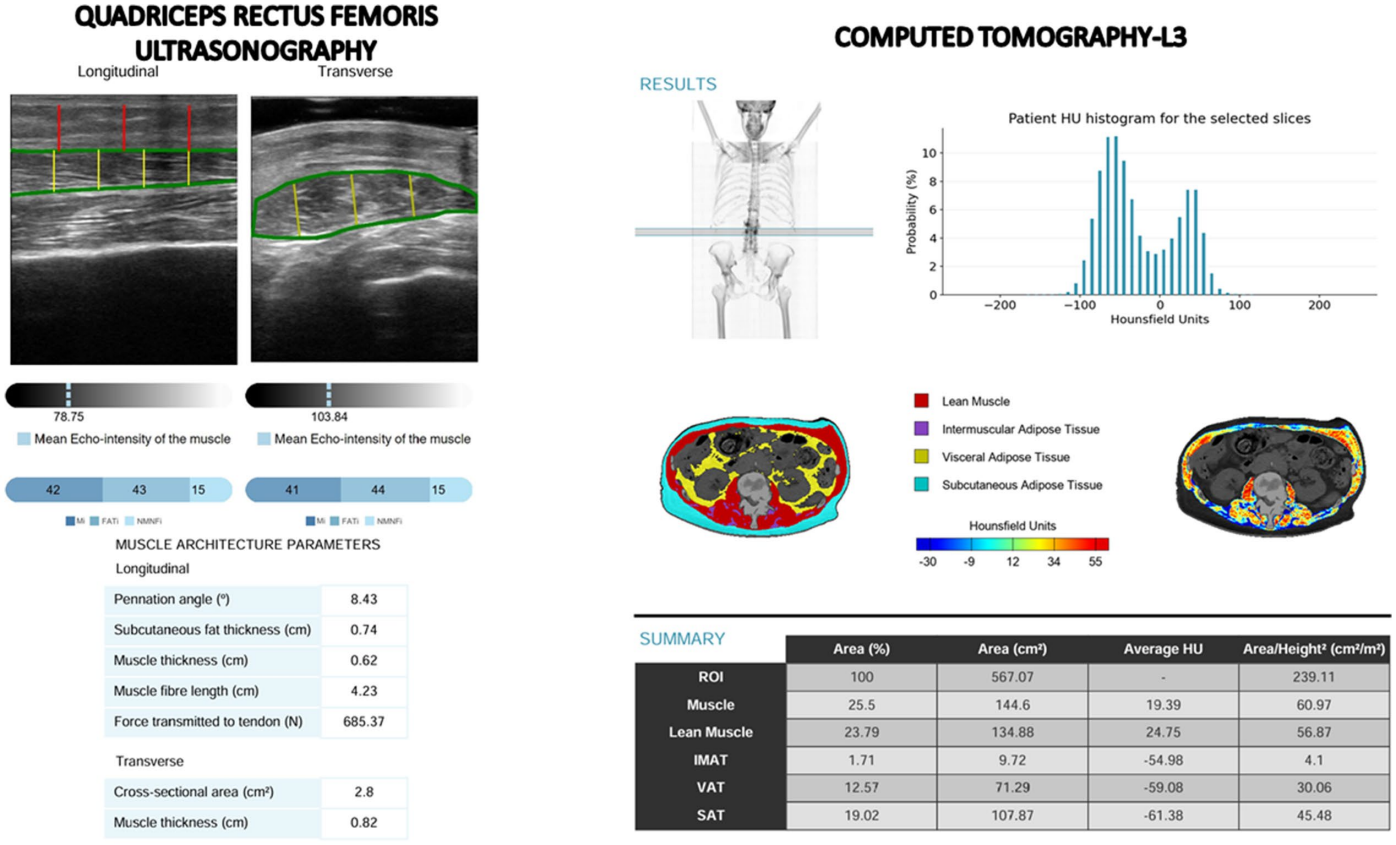
Images from quadriceps rectus femoris ultrasonography (PIIXMEDTM) and computed tomography-L3 (FocusedON) with artificial intelligence tools.

CT scans was centered on the third lumbar vertebra, averaging the slices between the upper and lower plates of the vertebra. Skeletal muscle was assessed using cross-sectional CT images at L3, evaluating the following muscle groups: psoas, erector spinae, quadratus lumborum, transversus abdominis, external obliques, internal obliques, and rectus abdominis, assessing Skeletal Muscle Area (SMA) in cm^2^, skeletal muscle index (SMI) in cm^2^/m^2^, and the average Hounsfield Unit (SM-HU). Adipose tissue was classified into subcutaneous (SAT), visceral (VAT), and intramuscular fat (IMF), with all areas measured in cm2. Tissue quality was determined based on its mean Hounsfield Unit (SAT-HU, VAT-HU, IMAT-HU) value. This tool can differentiate lean muscle area (LMA) from SMA as the content of muscle without IMF (Figure 1). Body composition components (SMA, LMA, IMF, VAT, SAT) were represented as well as the percentage from the ROI.

We have used a 2D approach to evaluate the muscle mass in the CT scans due to two main reasons: (1) Sarcopenia cut-off values most commonly used in oncology-such as those published by Martin et al. for mortality risk-are based on two dimensional CT measurements at the L3 vertebral level (18). To enable direct comparability with these established thresholds and ensure that our findings can be interpreted against the same benchmarks, we therefore performed all CT muscle assessments using identical 2D parameters at L3. (2) Our primary objective was a head-to-head comparison with rectus femoris ultrasound (a 2D modality). Adopting 2D thickness and area ensured methodological consistency between modalities.

#### 2.3.3 AI-based muscular ultrasonography

Ultrasonography of the QRF muscle was conducted on the dominant leg using a 10–12 MHz multifrequency linear array probe (Mindray Z60, Madrid, Spain). The patient was positions supine, and the probe was held perpendicular to the muscle along the transverse axis of the dominant leg, specifically at the lower third of the distance between the iliac crest and the upper border of the patella (19).

Ultrasound images were processed using an AI-driven imaging system (PIIXMED™; DAWAKO MedTech, Valencia, Spain) based on a convolutional neural network with a U-Net architecture. This system enables 2D feature extraction from conventional B-mode ultrasound by computing single-value metrics for each feature within a defined ROI. A range of biomarkers was derived by analyzing these features and applying various algorithms to assess the ROI’s morphological structure, muscle quality (via echogenicity), and multiple texture-based characteristics (16). The system has been trained on ultrasound images from healthy athletes as well as on individuals with pathologies that can affect muscle quality.

Muscle quality was assessed by measuring the pennation angle—the angle between the muscle fibers and the lower aponeurosis—with larger angles indicating greater potential for muscle strength. Additionally, muscle quality indices were computed using a multi thresholding algorithm (Multi-Otsu) that analyzed histogram echogenicity and grayscale intensity, applying defined thresholds to classify pixels into distinct tissue categories (20). Multi-Otsu refines the classic Otsu thresholding, which normally splits an image into foreground and background, by introducing multiple intensity cut-off points. Instead of just two classes, it partitions pixels into three or more groups, making it well suited for images with several ROI. The algorithm analyzes the histogram to find thresholds that minimize variance within each class while maximizing variance between classes. This algorithm produced three quantitative indices from transverse ultrasound images: the Muscle Index (MiT), representing the percentage of muscular tissue within the ROI; the Fat Index (FATi), indicating the proportion of intramuscular adipose tissue; and the No Muscle No Fat Index (NMNFiT), denoting the percentage of the ROI composed of other structural elements such as collagen, connective tissue, or fibrosis. All indices were expressed as percentages of the total ROI (Figure 1).

#### 2.3.4 Muscle strength

Muscle functionality was evaluated by measuring handgrip strength with a JAMAR® dynamometer (Basel, Switzerland). Patients were seated with their dominant arm positioned at a right angle to the forearm while performing the handgrip test.

#### 2.3.5 Nutritional diagnosis

- Malnutrition diagnosis: Malnutrition was diagnosed according the Global Leadership Initiative on Malnutrition (GLIM) criteria, which require the presence of at least one phenotypic criterion and one etiologic criterion (6). Low muscle mass phenotypic criterion was done with cut-off points of CT-L3 total muscle area adjusted by height, established by Martin et al. in patients with cancer (BMI > 25 kg/m^2^: men <53 cm2/m^2^, women <43cm^2^/m^2^; BMI < 25 kg/m^2^: men<43cm^2^/m^2^, women<41 cm^2^/m^2^) (18).
- Sarcopenia diagnosis: Sarcopenia was identified using the European Working Group on Sarcopenia in Older People (EWGSOP2) criteria. This diagnosis necessitated an impaired handgrip strength, defined as less than 27 kg in men and less than 16 kg in women, coupled with reduced muscle mass, with low muscle mass defined with the same cut-off points by Martin et al. (18). Patients exhibiting compromised handgrip strength without low muscle mass were classified as having probable sarcopenia or dynapenia.
- Myosteatosis diagnosis: Low muscle quality was defined using CT-derived skeletal muscle attenuation (SM-HU), based on the survival-related cut-off values established by Martin et al. (18) According to these criteria, myosteatosis is identified by an SM-HU value of less than 41 HU in patients with a BMI below 25 kg/m^2^, and less than 33 HU in patients with a BMI of 25 kg/m^2^ or higher.

### 2.4 Statistical analysis

Statistical analysis was performed using SPSS version 15.0 (SPSS Inc., Chicago, IL, USA), under an official license by the University of Valladolid. The Kolmogorov–Smirnov test assessed the normality of continuous variables. Variables following a normal distribution are presented as the mean (standard deviation), while those not normally distributed are reported as the median with interquartile range. Categorical variables are expressed as frequency (number and percentage of the total sample). For comparing parametric continuous variables, the unpaired Student’s T-test was used; in contrast, the Mann–Whitney *U*-test was applied for non-parametric data. Additionally, correlation analyses were conducted to determine the relationships between quantitative variables.

Furthermore, the diagnostic validity of the muscular ultrasound test was evaluated using ROC analysis for sarcopenia (diagnosed by EWGSOP with CT-L3 measurements). Cut-off points were determined through the application of the Youden index (Sensitivity + Specificity-1), which also allowed for the calculation of both the positive predictive value (PPV) and the negative predictive value (NPV).

## 3 Results

### 3.1 Sample description

A total of 337 oncology patients were included, with 198 (58.8%) men and a mean age of 69.7 (10.9) years. The distribution of cancer types was as follows: esophagogastric (34.4%), colorectal (25.8%), hepatobiliary-pancreatic (17.2%), urological (6.1%), lung (5.5%), head and neck (5.2%), gynecological (3.4%), breast (1.5%), and hematological (0.9%). There were difference in type of pathologies related to gender (Table 1).

**Table 1:** Differences between genders in baseline characteristics.

Variables	Total (*n* = 337)	Men (*n* = 198)	Women (*n* = 139)	*p*-value
Age (years)	69.7 (10.9)	69.98 (10.87)	69.24 (10.91)	0.54
Localization of cancer (%):				
Esophagogastric	34.4	38	29.1	<0.01
Colorectal	25.8	26.6	24.6	
Hepatobiliary-pancreatic	17.2	16.7	17.9	
Urological	6.1	8.3	3	
Lung	5.5	4.7	6.7	
Head and neck	5.2	4.7	6	
Gynecological	3.4	0	8.2	
Breast	1.5	0	3.7	
Hematological	0.9	1	0.7	
Anthropometry				
BMI (kg/m^2^)	23.69 (4.62)	23.84 (4.36)	23.49 (4.97)	0.5
Arm Circumference (cm)	25.37 (3.37)	25.63 (3.03)	24.97 (3.81)	0.09
Calf Circumference (cm)	32.59 (3.71)	32.98 (3.63)	32.05 (3.78)	0.03
Quadriceps rectus femoris ultrasonography				
RFA (cm^2^)	3.03 (0.99)	3.36 (0.99)	2.56 (0.78)	<0.01
RFI (cm^2^/m^2^)	1.15 (0.36)	1.22 (0.37)	1.05 (0.32)	<0.01
RFT (cm)	0.95 (0.26)	1.02 (0.26)	0.86 (0.22)	<0.01
MiT (%)	45.64 (9.64)	47.37 (9.86)	43.18 (8.78)	<0.01
FATi (%)	40.03 (6.27)	39.13 (6.95)	40.92 (6.19)	0.02
NMNFATi (%)	14.49 (4.62)	13.50 (4.42)	15.91 (4.56)	<0.01
MiArea (cm^2^)	1.41 (0.63)	1.61 (0.64)	1.12 (0.47)	<0.01
FATiArea (cm^2^)	1.19 (0.44)	1.31 (0.45)	1.04 (0.38)	<0.01
Pennation Angle (°)	5.51 (2.81)	5.80 (2.85)	5.05 (2.67)	0.02
Computed tomography				
*SMA (cm^2^)*	131.65 (28.24)	144.70 (25.47)	113.05 (20.57)	<0.01
*SMI (cm^2^/m^2^)*	49.39 (10.63)	51.76 (11.17)	45.99 (8.81)	<0.01
Muscle Attenuation (HU)	24.76 (12.41)	24.91 (11.61)	24.53 (13.51)	0.78
LMA (cm^2^)	119.96 (26.38)	132.34 (23.73)	102.33 (18.91)	<0.01
LMI (cm^2^/m^2^)	44.89 (9.59)	47.23 (10.05)	41.54 (7.79)	<0.01
Lean Muscle (% Muscle)	91.20 (5.44)	91.56 (4.96)	90.69 (6.03)	0.15
IMFA (cm^2^)	11.68 (7.68)	12.36 (7.74)	10.72 (7.51)	0.05
IMFI (cm^2^/m^2^)	4.49 (3.06)	4.53 (2.99)	4.45 (3.14)	0.83
Intramuscular Fat (% Muscle)	8.79 (5.44)	8.44 (4.96)	9.31 (6.03)	0.15
Muscle function				
Handgrip Strength (kg)	24.16 (8.38)	28.00 (7.32)	18.84 (6.69)	<0.01

BMI: Body Mass Index; RFA: Rectus Femoris Area; RFI: Rectus Femoris Index; RFT: Rectus Femoris Thickness; ROI: Region of Interest; MiT: ROI Muscle percentage; FATi: ROI Fat percentage; NMNFATi: ROI No muscle, no fat percentage; MiArea ROI Muscle Area; FATiArea: Roi Fat Area; SMA: Skeletal Muscle Area; SMI: Skeletal Muscle Index; HU: Houmsfield Units; LMA: Lean Muscle Area; LMI: Lean Muscle Index; IMFA: Intramuscular Fat Area; IMFI: Intramuscular Fat Index.

Body composition parameters are represented in Table 1, where we observed that BMI and arm circumference showed no significant gender differences, but men had notably larger calf circumference. Ultrasonography revealed that men had significantly greater quadriceps muscle area, thickness, and quality (higher MiT and lower NMNFATi), while women exhibited higher FATi. Computed tomography confirmed that men had larger skeletal and lean muscle areas, higher muscle indices, and slightly more intramuscular fat, though muscle attenuation was similar across sexes. Functionally, men demonstrated significantly stronger handgrip strength. Overall, men tended to have greater muscle mass and strength, while women showed higher fat infiltration in muscle tissue (Table 1).

### 3.2 Malnutrition and sarcopenia prevalence

**Figure 2:**
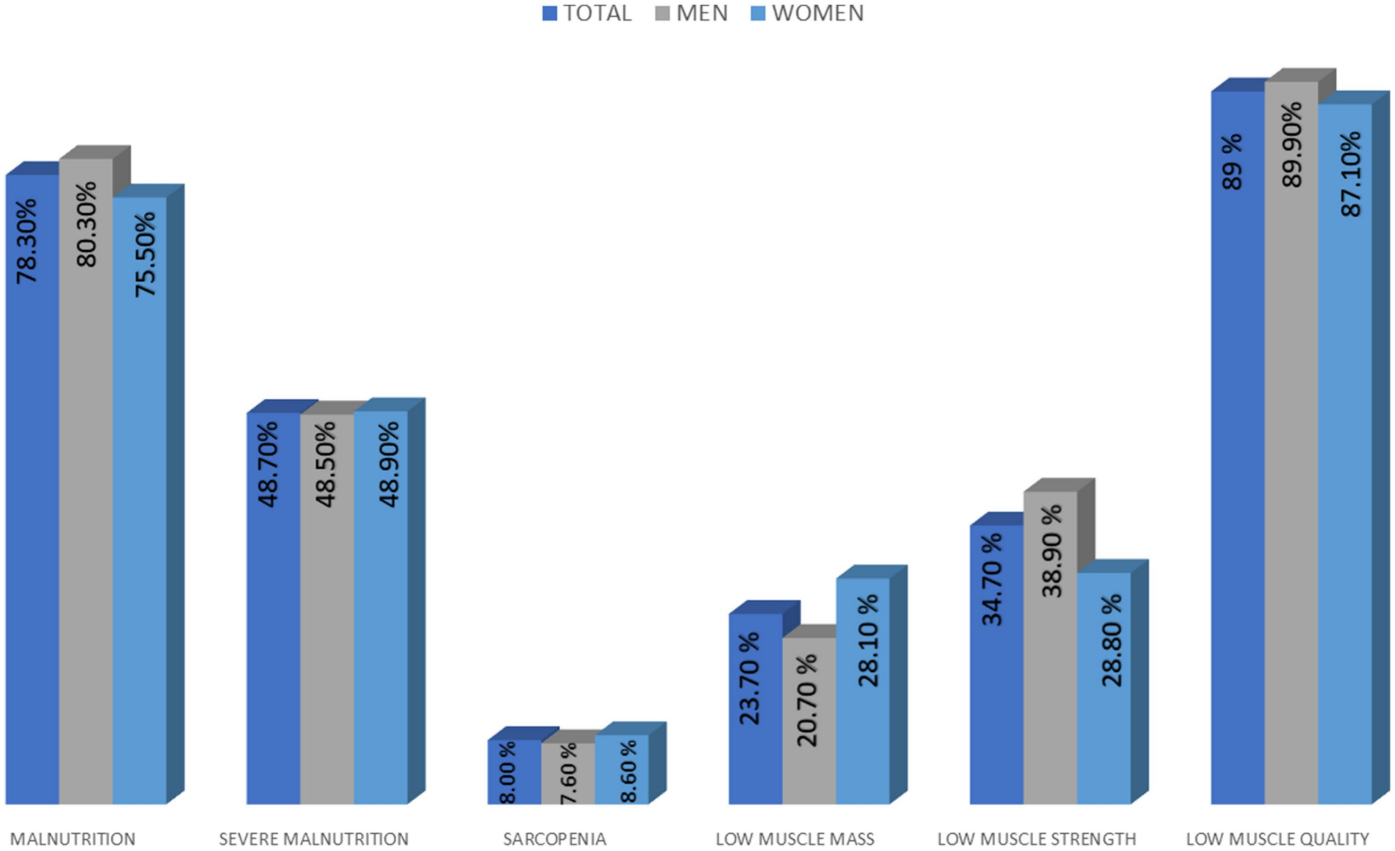
Diagnosis of malnutrition and sarcopenia and differences related to gender.

According to the GLIM criteria, malnutrition was present in 264 patients (78.3%), of whom 164 (62.1%) had severe malnutrition, accounting for 48.7% of the total sample (Figure 2). Sarcopenia was identified in 27 patients (8%). Additionally, 80 patients (23.7%) had low muscle mass (per Martin et al. cut-offs), and 117 (34.7%) had reduced muscle strength. Myosteatosis was prevalent in 299 patients (88.7%) (Figure 2). There were no significant differences related to gender in malnutrition (Men: 80.3%; women: 75.50%), sarcopenia (Men: 7.6%; women: 8.6%) or its components (Low muscle mass: Men: 20.70%; women: 28.10%; low muscle strength: Men: 38.90%; women: 28.90%; low muscle quality: Men: 89.90%; women: 87.10%) (Figure 2).

### 3.3 Muscle mass and quality assessment

Table 2 compares ultrasound muscle-related metrics across sarcopenia and its components (sarcopenia, low muscle mass, low muscle strength and low muscle quality) assessed by L3 CT; sarcopenia and low muscle mass are associated with significant reductions in muscle area, thickness, and pennation angle, while low muscle strength primarily impacts muscle tissue and area with increased fat infiltration. Low muscle quality, assessed via CT attenuation, shows the most pronounced decline in muscle tissue and area, alongside elevated fat indicators. Lower muscle quality parameters on ultrasound were associated with decreased CT attenuation values and reduced muscle strength. Across all conditions, most differences are statistically significant, underscoring the distinct physiological impacts of each (Table 2).

**Table 2:** Differences in ultrasonographic parameters between muscle mass and quality components of sarcopenia and diagnosis of sarcopenia using CT values.

Sarcopenia (Low SMI + Low Handgrip Strength)			
Variables	YES (*n* = 26)	NO (*n* = 274)	*p*-value
RFA (cm^2^)	2.66 (0.88)	3.05 (0.99)	0.05
RFI (cm^2^/m^2^)	1.00 (0.29)	1.15 (0.36)	0.03
RFT (cm)	0.85 (0.22)	0.96 (0.25)	0.03
MiT (%)	46.21 (9.85)	45.53 (9.73)	0.73
FATi (%)	41.24 (6.17)	39.90 (6.26)	0.30
NMNFATi (%)	14.92 (5.82)	14.45 (4.53)	0.62
MiArea (cm^2^)	1.25 (0.56)	1.41 (0.63)	0.21
FATiArea (cm^2^)	1.02 (0.36)	1.21 (0.44)	0.04
Pennation Angle (°)	4.37 (2.82)	5.61 (2.77)	0.03

Low muscle mass (Low SMI (Martin cut-off points))			
Variables	YES (*n* = 80)	NO (*n* = 255)	*p*-value
RFA (cm^2^)	2.73 (0.92)	3.12 (0.99)	*<0.01*
RFI (cm^2^/m^2^)	1.00 (0.32)	1.19 (0.35)	*<0.01*
RFT (cm)	0.86 (0.23)	0.98 (0.25)	*<0.01*
MiT (%)	39.20 (7.13)	40.28 (5.98)	0.06
FATi (%)	39.20 (7.13)	40.28 (5.98)	0.19
NMNFATi (%)	14.11 (5.85)	14.65 (4.16)	0.36
MiArea (cm^2^)	1.32 (0.63)	1.43 (0.62)	0.19
FATiArea (cm^2^)	1.03 (0.37)	1.25 (0.44)	*<0.01*
Pennation Angle (°)	4.89 (2.62)	5.69 (2.84)	0.03

Low muscle strength (Low Handgrip Strength)			
Variables	YES (*n* = 110)	NO (*n* = 190)	*p*-value
RFA (cm^2^)	2.89 (0.97)	3.10 (1.01)	0.07
RFI (cm^2^/m^2^)	1.12 (0.36)	1.16 (0.35)	0.44
RFT (cm)	0.92 (0.26)	0.97 (0.25)	0.11
MiT (%)	44.17 (9.47)	46.45 (9.79)	*0.04*
FATi (%)	41.29 (5.51)	39.28 (6.55)	*<0.01*
NMNFATi (%)	14.83 (4.84)	14.25 (4.53)	0.27
MiArea (cm^2^)	1.28 (0.54)	1.47 (0.67)	*0.01*
FATiArea (cm^2^)	1.18 (0.48)	1.20 (0.42)	0.73
Pennation Angle (°)	5.53 (2.94)	5.48 (2.71)	0.87

Low muscle quality (Low CT attenuation (Martin Cut-Off Points))			
Variables	YES (*n* = 299)	NO (*n* = 38)	*p*-value
RFA (cm^2^)	2.99 (0.99)	3.39 (0.99)	0.02
RFI (cm^2^/m^2^)	1.13 (0.36)	1.26 (0.34)	0.03
RFT (cm)	0.94 (0.26)	1.05 (0.24)	0.01
MiT (%)	45.01 (9.15)	50.59 (11.89)	<0.01
FATi (%)	40.19 (6.45)	37.31 (8.06)	<0.01
NMNFATi (%)	14.79 (4.45)	12.09 (5.27)	<0.01
MiArea (cm^2^)	1.36 (0.59)	1.75 (0.74)	<0.01
FATiArea (cm^2^)	1.19 (0.44)	1.25 (0.42)	0.44
Pennation Angle (°)	5.55 (2.78)	5.17 (3.02)	0.43

RFA: Rectus Femoris Area; RFI: Rectus Femoris Index; RFT: Rectus Femoris Thickness; ROI: Region of Interest; MiT: ROI Muscle percentage; FATi: ROI Fat percentage; NMNFATi: ROI No muscle, no fat percentage; MiArea ROI Muscle Area; FATiArea: Roi Fat Area. SMI Skeletal Muscle Index.

Among men, no significant differences in CT-derived muscle quality (SM-HU, LM-HU, VAT-HU, SAT-HU) were observed between those with and without sarcopenia. Similarly, no significant differences were found in women for SM-HU and LM-HU by sarcopenia status. However, women with sarcopenia exhibited significantly higher VAT-HU [−68.4 (15.8) vs. –81.9 (13.8); *p* < 0.01] and SAT-HU values [−74.1 (23.1) vs. –89.9 (20.6); *p* = 0.01].

### 3.4 Association with dynapenia

Men with dynapenia had significantly lower muscle density on CT compared to non-dynapenic individuals: SM-HU [21.1 (10.6) vs. 27.5 (11.7); *p* < 0.01] and LM-HU [30.6 (7.8) vs. 34.5 ± 8.4; *p* < 0.01]. In women, dynapenic participants also showed reduced SM-HU [17.7 (14.4) vs. 26.9 (12.0)] and LM-HU [28.8 (9.7) vs. 35.0 (8.6)], both with *p* < 0.01. No significant differences in VAT-HU or SAT-HU were observed between dynapenic and non-dynapenic groups in either sex.

### 3.5 Ultrasound-CT correlations

**Figure 3:**
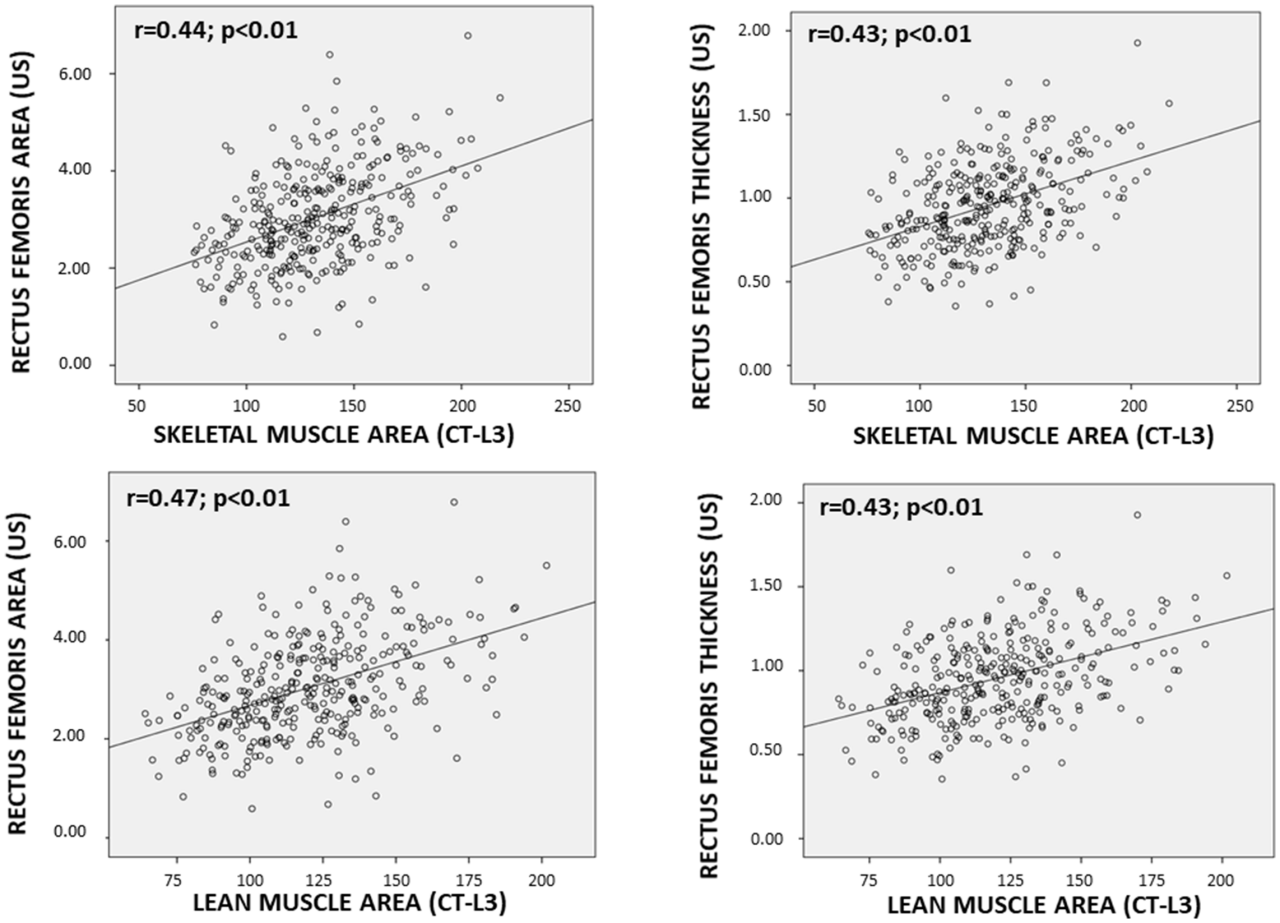
Scatter Plot between muscle mass parameters determined by ultrasonography (US) and computed tomography-L3 (CT-L3).

Ultrasound-derived muscle mass parameters (e.g., rectus femoris area) showed moderate correlations with CT-based skeletal muscle area (SMA) and lean muscle area (LMA) (Figure 3). Muscle quality indicators, such as MiT, demonstrated weak positive correlations with CT-based muscle quality measures, while FATi and NMNFiT showed negative correlations with muscle parameters and positive correlations with intramuscular fat content (Table 3).

**Table 3:** Correlation between ultrasound variables and computed tomography variables.

Variables	*SMA*	*SM-HU*	*LMA*	*LM-HU*	*%LM*	*IMFA*	*%IMF*
*RFA*	*r* = 0.44 *p* < 0.01	*r* = 0.14 *p* < 0.01	*r* = 0.47 *p* < 0.01	*r* = 0.13 *p* < 0.02	*r* = −0.13 *p* = 0.01	*r* = 0.03 *p* = 0.65	*r* = −0.15 *p* = 0.01
*RFT*	*r* = 0.43 *p* < 0.01	*r* = 0.08 *p* = 0.12	*r* = 0.43 *p* < 0.01	*r* = 0.10 *p* = 0.06	*r* = −0.06 *p* = 0.29	*r* = 0.09 *p* = 0.07	*r* = 0.06 *p* = 0.29
*MiT*	*r* = 0.02 *p* = 0.75	*r* = 0.27 *p* < 0.01	*r* = 0.09 *p* = 0.12	*r* = 0.25 *p* < 0.01	*r* = 0.25 *p* < 0.01	*r* = −0.23 *p* < 0.01	*r* = −0.25 *p* < 0.01
*FATi*	*r* = 0.09 *p* = 0.07	*r* = −0.21 *p* < 0.01	*r* = −0.01 *p* = 0.41	*r* = −0.19 *p* < 0.01	*r* = −0.22 *p* < 0.01	*r* = 0.19 *p* < 0.01	*r* = 0.14 *p* < 0.01
*NMNFATi*	*r* = −0.10 *p* = 0.07	*r* = −0.26 *p* < 0.01	*r* = −0.16 *p* < 0.01	*r* = −0.16 *p* < 0.01	*r* = −0.24 *p* < 0.01	*r* = 0.19 *p* < 0.01	*r* = 0.24 *p* = 0.01
*Pennation Angle*	*r* = 0.13 *p* < 0.02	*r* = 0.02 *p* = 0.73	*r* = 0.12 0.03	*r* = 0.05 *p* = 0.34	*r* = −0.02 *p* = 0.69	*r* = 0.07 *p* = 0.19	*r* = 0.02 *p* = 0.69

Red: Very weak; yellow: weak; blue: moderate; green: strong. RFA: Rectus Femoris Area; RFT: Rectus Femoris Thickness; ROI: Region of Interest; MiT: ROI Muscle percentage; FATi: ROI Fat percentage; NMNFATi: ROI No muscle, no fat percentage; SMA: Skeletal Muscle Area; SM-HU: Hounsfield Units Skeletal Muscle; LMA: Lean Muscle Area; LM-HU: Hounsfield Units Lean Muscle; %LM: percentage Lean Muscle; IMFA: Intramuscular Fat Area; %IMF: percentage Intramuscular Fat.

### 3.6 Diagnostic performance and cut-off points

**Figure 4:**
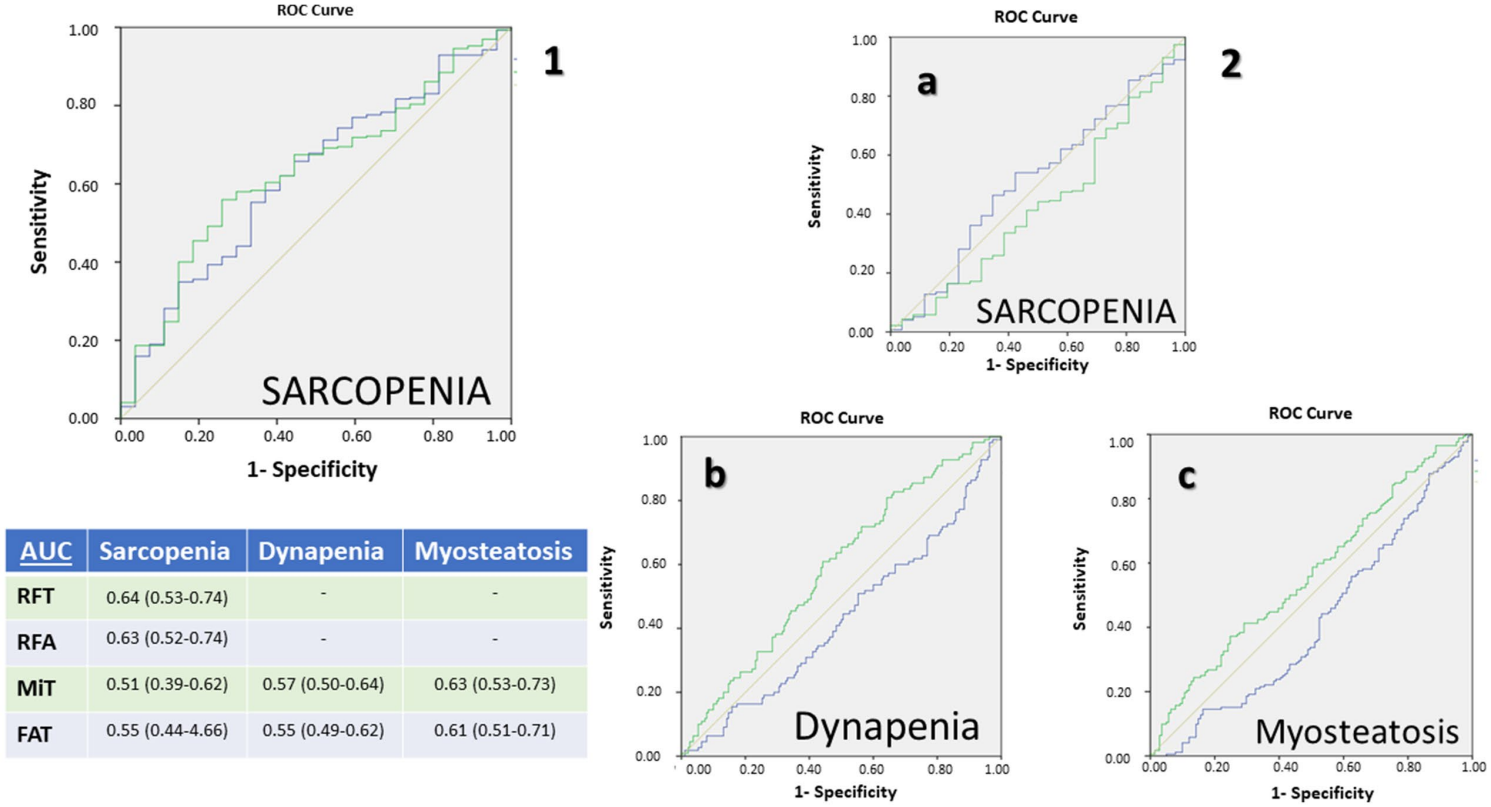
Receiving operating characteristic (ROC) curves for muscular ultrasonography of rectus femoris: **(1)** Muscle mass parameters: rectus femoris muscle area (blue) and rectus femoris thickness (green) for the diagnosis por sarcopenia; **(2)** Muscle quality parameters: percentage of muscle mass (Mi) (green) and percentage of fat mass (FATi) (blue) for the diagnosis of sarcopenia **(a)**, dynapenia **(b)**, and low muscle quality **(c)**. AUC: Area Under Curve.

Receiver operating characteristic (ROC) analyses for ultrasound-based markers are summarized in Table 4 and Figure 4. For sarcopenia detection, rectus femoris thickness (RFT) yielded an AUC of 0.64 (95% CI 0.53–0.74), and rectus femoris area (RFA) 0.63 (95% CI 0.52–0.74).

**Table 4:** Cut-off points of Artificial Intelligence enhanced ultrasound variables of the rectus femoris for detecting sarcopenia and its components (muscle mass and quality) in all patients and distributed by sex.

Variables	Categories	Patients	Cut-Off Value	AUC	Sensitivity (%)	Specificity (%)	PPV (%)	IC95%	NPV (%)	IC95%
Rectus femoris area (cm^2^)	Sarcopenia	All	2.845	0.628	55.25	66.67	12.60	6.6–18.6	94.49	91.5–97.5
		Men	3.425	0.598	49.40	73.30	65.00	55.46–65.40	59.20	52.90–65.40
		Women	2.845	0.632	34.10	100	100	69.20–100	94.60	92.15–97.05
	Low muscle strength	All	3.09	0.565	46.90	65.80	46.09	37.5–54.7	66.53	60.10–73
		Men	3.221	0.616	62.20	57.10	47.40	39.9–54.9	70.79	63.9–77.7
		Women	2.801	0.582	37.50	77.50	40.93	30.8–51.1	71.86	66.3–77.5
	Low muscle mass	All	2.71	0.619	64.70	55.00	30.87	23.8–37.9	83.38	77.5–89.3
		Men	2.71	0.580	78.80	36.60	24.50	18.8–30.2	86.87	80.8–92
		Women	1.88	0.617	88.90	30.80	33.43	27.5–39.3	87.65	80.6–94.4
	Low muscle quality	All	3.68	0.624	47.40	78.60	95.15	91.6–98	23.07	15.7–0.30.8
		Men	3.68	0.654	65.00	68.00	94.53	91–98	27.81	18–37.7
		Women	2.64	0.667	61.10	63.60	91.80	87.5–96.1	19.50	13–26
Rectus femoris thickness (cm)	Sarcopenia	All	0.912	0.636	54.90	74.10	15.6	8.31–22.89	94.97	92.22–97.72
		Men	1.180	0.613	25.60	93.30	25.00	8.9–41.10	93.50	90.8–96.30
		Women	0.868	0.641	43.9	100	100	75.30–100	95.30	93–97.70
	Low muscle strength	All	0.901	0.557	60.50	53.60	37.49	30.5–44.5	68.85	61.9–75.7
		Men	0.924	0.580	73.80	47.30	47.13	40.3–54.9	73.93	66.5–81.3
		Women	0.895	0.578	42.20	72.20	38.04	28.9–47.1	75.54	69.8–81.3
	Low muscle mass	All	0.888	0.619	61.60	63.70	34.52	26.8–42.2	84.23	79–89.4
		Men	1.086	0.593	39.10	78.00	11.57	7.3–15.3	58.05	48.3–67.7
		Women	0.867	0.633	49.50	84.60	55.68	44.9–66.3	81.08	76.2–86
	Low muscle quality	All	0.96	0.631	71.10	54.80	92.50	89–96	19.40	11.6–27.2
		Men	0.95	0.633	85.00	42.70	93.00	90–96	24.20	15.3–33.1
		Women	1.13	0.680	38.90	90.01	96.40	93.1–99.7	17.90	12.7–23.1
Mi (%)	Sarcopenia	All	43.32	0.505	52.50	36.60	6.72	3.34–10.10	89.86	84.59–95.13
		Men	44.69	0.416	54.70	46.70	7.78	3.87–11.69	92.60	88.51–96.69
		Women	47.29	0.602	30.10	100	100	66.39–100	93.83	91.24–96.42
	Low muscle strength	All	40.62	0.569	76.80	37.30	39.43	33–45.8	75.16	66.9–83.4
		Men	51.02	0.555	36.40	76.70	49.86	39.9–59.9	65.45	59.3–71.6
		Women	40.61	0.645	77.10	55.60	41.26	34.1–48.5	85.72	79–91.5
	Low muscle mass	All	62.68	0.547	12.50	97.30	58.98	35.1–82.9	78.17	73.7–83.7
		Men	46.45	0.629	65.90	57.70	28.91	21.9–36.9	86.63	81.4–91.1
		Women	34.64	0.491	94.90	84.80	70.93	62.9–78.9	97.70	95.4–100
	Low muscle quality	All	46.77	0.640	68.40	63.90	93.70	90.3–97.1	20.50	13.1–27.9
		Men	47.72	0.691	75.00	62.40	94.70	91.9–97.5	21.90	13.5–30.3
		Women	46.73	0.614	61.10	73.60	94.00	90.5–97.5	21.90	15–28.8
FATi (%)	Sarcopenia	All	38.11	0.549	66.40	48.10	9.91	5.53–14.29	96.55	93.7–99.40
		Men	38.11	0.603	63.40	60.00	11.14	5.94–16.34	96.37	93.75–98.99
		Women	47.16	0.479	13.80	100	13.80	1.2–26.4	92.03	88.90–95
	Low muscle strength	All	40.40	0.555	59.00	45.40	32.71	26–39.5	63.45	55.6–71.2
		Men	36.68	0.547	63.10	12.20	29.23	23.8–34.7	32.66	21.9–43.4
		Women	40.41	0.602	72.50	56.20	43.47	35.8–51.2	62.16	54.7–69.7
	Low muscle mass	All	41.25	0.567	44.70	67.50	27.57	19.6–35.6	77.47	71.8–83.2
		Men	37.61	0.614	67.30	51.20	16.04	10.5–21.6	59.02	53.1–65
		Women	46.17	0.523	19.20	92.30	41.43	31–51.8	77.25	72.5–82.1
	Low muscle quality	All	41.20	0.623	45.50	78.90	94.40	90.5–98.3	15.60	10.4–20.8
		Men	41.14	0.642	44.40	80.00	95.20	91.8–98.6	13.90	8.9–18.9
		Women	38.66	0.622	71.10	61.61	92.90	89.4–96.4	23.20	15.3–31.1

AUC: Area Under Curve; PPV: Positive Predictive Value; NPV: Negative Predictive Value. MiT: ROI Muscle percentage; FATi: ROI Fat percentage.

For muscle quality assessed by AI-assisted ultrasonography, the MiT achieved its highest performance in identifying myosteatosis with an AUC of 0.63 (95% CI 0.53–0.73), followed by an AUC of 0.57 (95% CI 0.50–0.64) for low muscle strength and 0.51 (95% CI 0.39–0.62) for sarcopenia. The FiT performed best for myosteatosis (AUC 0.61, 95% CI 0.51–0.71) and showed lower concordance for low muscle strength (AUC 0.55, 95% CI 0.49–0.62) and sarcopenia (AUC 0.55, 95% CI 0.44–0.66).

Sex-specific thresholds were identified. In the overall population, a cross-sectional area threshold of 2.845 cm^2^ had high specificity in women, while RFT demonstrated a higher threshold in men with strong negative predictive value. Notably, Mi and FATi indices showed distinct diagnostic thresholds between sexes, emphasizing the need for sex-specific cut-offs tailored to the condition being assessed (Table 4).

The application of these cut-off points to select quality parameters and establish diagnostic criteria for muscle mass and muscle quality based on MiT and FiT, stratified by gender, yielded the following findings. MiT-based diagnosis of low muscle mass showed significant differences in muscle area and muscle attenuation, both in total muscle and in the percentage of lean mass. In contrast, FiT-based diagnosis of low mass detected significant differences only in intramuscular fat area and intramuscular fat attenuation. Regarding muscle quality, both MiT and FiT criteria for low muscle quality identified significant differences in the percentages of total muscle, lean muscle, and intramuscular fat, as well in muscle attenuation and lean muscle attenuation (Table 5).

**Table 5:** Differences between L3 Computed Tomography based on cut-off points for sarcopenia in ultrasound muscle quality parameters.

Low muscle mass by MiT (Cut-Off points: MiT Men: 46.45%; MiT Women: 34.64%)				Low muscle mass by FiT (Cut-Off points: FiT Men: 37.61%; FiT Women: 46.17%)			
Variables	YES (*n* = 143)	NO (*n* = 194)	*p*-value	Variables	YES (*n* = 31)	NO (*n* = 306)	*p*-value
SMA (%ROI)	22.65 (4.80)	22.55 (5.06)	0.85	SMA (%ROI)	21.35 (5.16)	22.72 (4.91)	0.14
SMI (cm2/m2)	52.89 (8.93)	47.55 (9.44)	< 0.01	SMI (cm2/m2)	51.49 (9.11)	49.66 (9.63)	0.31
Muscle attenuation (HU)	22.69 (12.15)	26.27 (12.41)	< 0.01	Muscle attenuation (HU)	21.04 (14.94)	25.13 (12.09)	0.08
LMA (%ROI)	20.65 (5.07)	20.76 (5.31)	0.84	LMA (%ROI)	19.23 (5.57)	20.86 (5.15)	0.09
LMI (cm2/m2)	47.85 (7.96)	43.39 (8.64)	< 0.01	LMI (cm2/m2)	45.78 (7.91)	45.25 (8.71)	0.74
Lean muscle attenuation (HU)	31.44 (8.32)	34.36 (8.96)	< 0.01	Lean muscle attenuation (HU)	31.29 (9.39)	33.31 (8.73)	0.23
IMFA (%ROI)	2.00 (1.20)	1.79 (1.08)	0.08	IMFA (%ROI)	2.12 (1.39)	1.86 (1.11)	0.22
IMFI (cm2/m2)	5.05 (3.35)	4.09 (2.76)	< 0.01	IMFI (cm2/m2)	5.71 (4.29)	4.37 (2.88)	0.02
Intramuscular fat attenuation (HU)	−64.68 (5.94)	−63.91 (6.1)	0.24	Intramuscular fat attenuation (HU)	−66.90 (6.39)	−63.97 (5.95)	0.01

Low muscle quality by MiT (Cut-Off points: MiT Men: 47.72%; MiT Women: 46.73%)				Low muscle quality by FiT (Cut-Off points: FiT Men: 41.14%; FiT Women: 38.66%)			
Variables	YES (*n* = 212)	NO (*n* = 125)	*p*-value	Variables	YES (*n* = 176)	NO (*n* = 161)	*p*-value
SMA (%ROI)	21.68 (4.35)	24.15 (5.49)	< 0.01	SMA (%ROI)	21.64 (4.69)	23.63 (5.02)	<0.01
SMI (cm2/m2)	50.46 (9.20)	48.73 (10.15)	0.11	SMI (cm2/m2)	50.15 (9.02)	49.48 (10.19)	0.53
Muscle attenuation (HU)	22.23 (11.94)	29.05 (12.04)	< 0.01	Muscle attenuation (HU)	22.19 (12.17)	27.56 (12.09)	<0.01
LMA (%ROI)	19.68 (4.55)	22.47 (5.75)	< 0.01	LMA (%ROI)	19.65 (4.87)	21.88 (5.31)	<0.01
LMI (cm2/m2)	45.49 (8.23)	44.96 (9.31)	0.59	LMI (cm2/m2)	45.20 (8.16)	45.39 (9.15)	0.84
Lean muscle attenuation (HU)	31.28 (8.27)	36.26 (8.82)	< 0.01	Lean muscle attenuation (HU)	31.26 (8.29)	35.16 (8.91)	<0.01
IMFA (%ROI)	2.00 (1.15)	1.67 (1.09)	0.01	IMFA (%ROI)	1.99 (1.19)	1.75 (1.06)	0.05
IMFI (cm2/m2)	4.98 (3.17)	3.68 (2.67)	<0.01	IMFI (cm2/m2)	4.94 (3.24)	4.00 (2.76)	<0.01
Intramuscular fat attenuation (HU)	−64.65 (5.71)	−63.53 (6.53)	0.100	Intramuscular fat attenuation (HU)	−64.77 (5.32)	−63.65 (6.71)	0.09

ROI: Region of Interest; MiT: ROI Muscle percentage; FATi: ROI Fat percentage; SMA: Skeletal Muscle Area; SMI: Skeletal Muscle Index; HU: Hounsfield Unit; LMA: Lean Muscle Area; LMI: Lean Muscle Index; IMFA: Intramuscular Fat Area; IMFI: Intramuscular Fat Index.

## 4 Discussion

The AI powered Rectus Femoris Muscle ultrasonography is a valuable bedside tool for assessing muscle mass and quality. Comparisons between muscle ultrasonography and body composition parameters obtained from AI assisted CT at the L3 level showed moderate correlations for muscle mass indicators (RFA and RFT). In contrast, muscle quality parameters assessed by ultrasound (Mi, FATi, and NMNFATi) demonstrated weak but statistically significant correlations with CT-derived quality metrics (SM-HU, LM-HU, %LM, and %IMF). For diagnosing sarcopenia, ultrasound-based muscle mass parameters exhibited better AUC values. However, when diagnosis was based on muscle quality and strength, ultrasound-derived quality parameters showed superior predictive performance. Specifically, ultrasonographic assessment of muscle mass (low muscle mass) had higher negative predictive value, while muscle quality assessment (myosteatosis) had higher positive predictive value.

Several conditions can lead to sarcopenia, and the different diagnostic domains significantly influence the progression of muscle decline. In our study, we considered muscle quality as the initial stage of muscle deterioration. Accordingly, most patients in our sample exhibited low muscle quality, based on the myosteatosis values established by Martin et al. (41 HU for BMI < 25 kg/m^2^; and 33 HU for BMI > 25 kg/m^2^) (18). Other studies reported similar attenuation values (38.5 HU) in healthy kidney donor candidates. However, their study did not differentiate based on BMI, which may affect diagnostic accuracy (20). In contrast, Van der Werf et al. reported lower 5th percentile values for muscle attenuation – 29.3 HU for and 22 HU for women – which may reflect lower values with higher BMI (9). These discrepancies may be influenced by differences in population characteristics, as well as by the distinction between using a statistical metric such as a percentile and a clinical cut-off value. For instance, Martin’s study focused on cancer patients, while Van der Werf’s study involved healthy individuals. When applying Martin’s cut-off values, our ultrasound-based assessments showed poorer results in both muscle mass and muscle quality parameters.

On the other hand, the definition of low muscle mass varies across studies, and it is influenced by the selected cut-off points. The mean SMI in our sample (51.76 cm^2^/m^2^ for men and 45.99 cm^2^/m^2^ for women) was higher than that reported by Van der Werf et al., although their study included a non-oncological population with a wide age range (9). When compared to other cancer populations, such as in the study Martin et al., our mean values were similar, but the prevalence of low muscle mass was higher in their cohort (53% for women and 31% in men), likely due to the fact that their patients were in stage IV of cancer (18). Borrelli et al., in a small sample of cancer patients, reported even higher mean SMI values than ours, including among those diagnosed with sarcopenia (21). In that study, the cut-off points for low muscle mass were based on the Fearon et al. consensus, with a reported prevalence of 47.4% (22). Similarly, Faron et al., in a study of melanoma patients, found mean values comparable to ours, but defined low muscle mass using the median of their sample as the cut-off (23). The higher prevalence of low muscle mass in these studies, compared to ours (20.7% in men and 28.1% in women); may be attributed to differences in definitions and patient characteristics. However, it is important to note that many studies refer to sarcopenia solely based on low muscle mass, without considering muscle function (8).

Sarcopenia is defined primarily by low muscle strength, followed by low muscle mass. In our sample, only 8% of patients met the criteria for confirmed sarcopenia. However, when considering low muscle strength alone (probable sarcopenia), the prevalence increased to 34.7%. When compared with other studies using different assessment techniques, such as bioelectrical impedance analysis (BIA), the DRECO study reported a similar prevalence of confirmed sarcopenia (9.7%) but a lower rate of probable sarcopenia (14%) (13). In contrast, another study from our group, which included 68.4% oncologic patients, found a higher prevalence of sarcopenia at 20% (24). Patients with confirmed sarcopenia showed lower values in ultrasonographic muscle mass parameters and pennation angle, which reflects contractile capacity. Similarly, patients with low muscle strength exhibited altered values in ultrasonographic muscle quality parameters. These findings highlight the importance of muscle quality in relation to muscle function, suggesting that impairments in muscle quality may precede the decline in muscle mass (12).

Muscle mass parameters obtained via ultrasonography, specifically rectus femoris area (RFA) and thickness (RFT), showed moderate correlations with muscle mass parameters derived from AI-based CT imaging, such as skeletal muscle area (SMA) and lean muscle area. Jiménez-Sánchez et al. reported higher correlations with L3-SMA for RFT (*r* = 0.64) and RFA (*r* = 0.60), which may be attributed to the use of a homogeneous population consisting of colorectal cancer patients (25); In contrast, a study by Guirado et al. found a lower correlation for RFT and a higher one for muscle area. This discrepancy could be due to the partial relationship between muscle thickness and muscle function, whereas muscle area is more directly related to muscle mass (26). Nevertheless, the strongest correlations between techniques are observed when comparing parameters directly related to muscle mass. However, these correlations are not particularly strong, likely because they involve comparisons between trunk muscle mass and appendicular muscle mass (i.e., from the leg) at a single point. A correlation with dual-energy X-ray absorptiometry (DXA) might provide more accurate results.

The assessment of muscle quality, particularly the detection of atrophy or myosteatosis, is a key aspect in patient characterization using artificial intelligence. In this context, AI enables a more precise and standardized evaluation of fat infiltration in skeletal muscle through various tools applied across different imaging modalities, including CT, ultrasound or MRI (27). Numerous studies have demonstrated a relationship between muscle attenuation and overall patient prognosis in individuals who have undergone CT imaging (28). In fact, among oncology patients, lower muscle attenuation values and greater fat infiltration in skeletal muscle have been associated with poorer prognosis (27). When comparing muscle quality parameters assessed by ultrasonography, we observed weak correlations between the percentage of muscle and fat mass, muscle attenuation of total and lean muscle mass, and the percentage of muscle and fat within the ROI. The correlations were stronger when using the Muscle Index (Mi). Evaluation of muscle quality via ultrasonography has been associated with phase angle and muscle strength, as higher echogenicity values are linked to lower phase angle and reduced muscle strength in patients with DRM (11). Nevertheless, there are no specific studies directly comparing muscle quality measured by ultrasonography with that assessed by computed tomography. The assessment of myosteatosis using muscle ultrasonography is based on tissue echogenicity and the analysis of grayscale values to differentiate the proportion of muscle mass and fat mass within the defined ROI (29, 30). However, there are currently no well-validated reference values or diagnostic thresholds that allow us to determine prognostic implications. Additionally, the low correlation observed in our sample may be attributed to differences in muscle location and the specific ROI used for analysis.

Cut-off values for muscle mass (RFA and RFT) in sarcopenia were similar to those reported in the DRECO study, which used ASMI assessed by BIA to diagnose low muscle mass and applied the same cut-off points for handgrip strength (13). This is noteworthy because the DRECO study employed diagnostic criteria for primary sarcopenia, whereas our study used criteria for low muscle mass based on mortality risk, as proposed by Martin et al. (18). In our study, these cut-off values demonstrated better negative predictive values, consistent with findings from the DRECO study. This may be attributed to the use of extreme cut-off points for diagnosing sarcopenia, specifically, muscle mass thresholds based on mortality risk and handgrip strength values below the 5th percentile in patients over 75 years of age (8). This condition can influence over the worse ROC curves of association compared with other test as ASMI measured by BIA, like the study of De Luis et al. where AUC were 0.714 [0.785 for males and 0.813 for females (31)].

Ultrasonography-based muscle quality parameters showed superior performance when using cut-off points related to muscle quality, such as muscle attenuation on CT. These parameters yielded better positive predictive values for both Mi and FATi. Similar findings were reported by Kim et al. in a study involving patients undergoing hip surgery, where muscle quality was assessed using Sono elastography. In that study, sensitivity, specificity, and accuracy based on muscle attenuation were 77.3, 100, and 87.5%, respectively (32). The muscle quality values observed in our study may be linked to the high prevalence of myosteatosis detected among the participants.

This study has several limitations. First, the heterogeneity of the oncologic population, encompassing various tumor types, disease stages, and treatment modalities, may limit the generalizability of the findings despite efforts to standardize the timing of imaging. Second, the study was conducted at a single center, which may affect external validity. Third, although AI-assisted ultrasound allowed for standardized and automated assessment of muscle quality, the derived echogenicity-based biomarkers (Mi, FATi, NMNFi) lack external validation and remain specific to the technology and algorithms used. Fourth, muscle mass was assessed using two dimensional CT rather than 3D volumetric metrics; this choice aligns with established oncology sarcopenia thresholds (e.g., Martin et al.) and mirrors the inherently 2D nature of the rectus femoris ultrasound comparator. Lastly, the moderate correlations observed between ultrasound and CT may reflect the intrinsic anatomical and methodological differences between trunk-based and limb-based imaging assessments.

The principal strength of this study lies in its large, well-characterized cohort of oncology outpatients evaluated through a multimodal approach combining AI-enhanced CT, muscle ultrasound, and functional measures. Unlike studies focused solely on imaging-derived body composition, this research incorporates all core sarcopenia domains—muscle mass, quality, and strength—providing a comprehensive morphofunctional assessment. The use of deep learning-based segmentation tools for both imaging modalities improves measurement reproducibility and reduces operator dependence. Additionally, the definition of diagnostic cut-off points by sex offers clinically relevant benchmarks for sarcopenia assessment in nutritional oncology practice.

Future research should aim to validate ultrasonography cut-off points as prognostic markers in patients with oncologic conditions. This study may serve as a first step toward establishing ultrasonography as a simple, bedside tool for assessing muscle mass and quality during nutritional consultation in oncology patients. Additionally, AI-driven analysis of CT images could support the validation of these cut-off points in other populations affected by DRM, using opportunistic or even low-dose CT scans to evaluate body composition. Future studies should derive and validate also 3D volumetric cut-offs to enhance accuracy, reproducibility, and clinical utility.

## 5 Conclusion

This study demonstrates that artificial intelligence-assisted muscle ultrasonography of the rectus femoris is a feasible, non-invasive, and clinically relevant tool for assessing sarcopenia in oncology patients. It provides moderate correlations with gold-standard CT-based measurements of muscle mass and captures qualitative alterations related to muscle quality and fat infiltration. The identified sex-specific ultrasonographic cut-off points offer diagnostic value, with high negative predictive value for muscle mass and positive predictive value for myosteatosis. These findings support the integration of ultrasound, particularly when enhanced by AI segmentation, as a complementary bedside method in routine nutritional assessment. Future validation studies are warranted to confirm these cut-offs and to explore their prognostic implications in broader oncologic populations.

Advances in AI-driven body composition analysis are transforming clinical imaging. Deep learning is enhancing the precision of tissue segmentation and quantification, enabling not only accurate measurement of muscle and fat but also assessment of tissue quality, such as detecting fat infiltration and changes in density. Radiomics adds further value by extracting image-based patterns to build predictive models that link imaging biomarkers with clinical outcomes. Additionally, combining multiple imaging techniques may lead to more comprehensive 3D models, improving diagnosis and treatment planning.

## Data Availability

The raw data supporting the conclusions of this article will be made available by the authors, without undue reservation.
